# A Genome-Wide Search for Greek and Jewish Admixture in the Kashmiri Population

**DOI:** 10.1371/journal.pone.0160614

**Published:** 2016-08-04

**Authors:** Jonathan M. Downie, Tsewang Tashi, Felipe Ramos Lorenzo, Julie Ellen Feusier, Hyder Mir, Josef T. Prchal, Lynn B. Jorde, Parvaiz A. Koul

**Affiliations:** 1 Department of Human Genetics, University of Utah School of Medicine, Salt Lake City, Utah, United States of America; 2 Division of Hematology and Hematologic Malignancies, Department of Internal Medicine, University of Utah School of Medicine, Salt Lake City, Utah, United States of America; 3 Department of Internal & Pulmonary Medicine, Sher-i-Kashmir Institute of Medical Sciences, Srinagar, Jammu and Kashmir, India; Universitat Pompeu Fabra, SPAIN

## Abstract

The Kashmiri population is an ethno-linguistic group that resides in the Kashmir Valley in northern India. A longstanding hypothesis is that this population derives ancestry from Jewish and/or Greek sources. There is historical and archaeological evidence of ancient Greek presence in India and Kashmir. Further, some historical accounts suggest ancient Hebrew ancestry as well. To date, it has not been determined whether signatures of Greek or Jewish admixture can be detected in the Kashmiri population. Using genome-wide genotyping and admixture detection methods, we determined there are no significant or substantial signs of Greek or Jewish admixture in modern-day Kashmiris. The ancestry of Kashmiri Tibetans was also determined, which showed signs of admixture with populations from northern India and west Eurasia. These results contribute to our understanding of the existing population structure in northern India and its surrounding geographical areas.

## Introduction

The Kashmiri population is an Indo-European ethno-linguistic group from Jammu and Kashmir state in northern India. The precise origins of the Kashmiri population are unknown. It has been suggested that they are descendants of one of the “lost tribes” of Israel who were exiled in 722 BCE [1]. They are believed to have traveled along the Silk Road into the countries of the Middle East, Persia, and Afghanistan until they reached the Kashmir Valley and settled there [2]. The claim of Israelite ancestry is widespread among Kashmiris, who cite historical records and the similarity of geographical names and cultural and social traditions.

It has also been proposed that many of the rural tribes of Kashmir are of Greek descent as a result of the conquests of Alexander the Great [3]. It is thought that many of Alexander’s conscripts and soldiers settled in parts of India, including Kashmir, and intermixed with the local population once his conquests ended in India. This hypothesis has been supported by archeological evidence of ancient Greek presence in Kashmir [3]. For instance, a number of ancient Greek coins that date from shortly after the time of Alexander the Great’s presence in India have been found in Kashmir [3]. Furthermore, there is evidence that a number of Grecian words have been adopted into the local Kashmiri vernacular [3, 4]. There were also multiple Indo-Greek kingdoms, which were the remnants of further Hellenistic conquests into India, that ruled in what is now modern-day northern India, Pakistan, and Afghanistan [5]. The rule of the Indo-Greek kingdoms began in the Second Century BC and continued until the early First Century AD, furthering the notion of a substantial ancient Greek influence in Kashmir. It is thus reasonable to hypothesize that the current Kashmiri population may possess significant Greek ancestry.

To date, no genome-wide analyses have been performed to determine the degree to which Jewish or Greek genetic contributions exist in the Kashmiri population. The identification of such genetic admixture in the people of Kashmir would help to elucidate the population history of the region. It will also help to shed light on the history of nearby populations, such as the Pathans of Afghanistan and Pakistan, which also have been suggested to be of Jewish or Greek origin [1, 6]. One of the primary goals of this study is to determine whether detectable Greek or Sephardic Jewish genetic admixture is seen in individuals of Kashmiri descent using genome-wide genotyping assays. To our knowledge, this is the first attempt to answer this question using genome-wide genotype data.

## Methods

DNA was collected from 15 Kashmiri individuals from the Kashmir Valley who provided written consent. The collection and study of DNA from these individuals was approved by institutional review boards at the Sher-i-Kashmir Institute of Medical Sciences and the University of Utah School of Medicine (IRB 00017665). The families of these individuals have resided in the Kashmir Valley for at least three generations and have no history of marriages outside of the valley or to a non-Kashmiri. These individuals were then genotyped for single nucleotide polymorphisms (SNPs) using the Affymetrix Genome-Wide Human SNP Array 6.0. Sixteen Kashmiri Tibetans from Srinagar, India, who have Tibetan ancestry and now practice Islam, were also genotyped. We performed further genotyping on a previously undescribed population of 32 first- and second-generation Tibetan exiles in McLeod Ganj, which included two individuals from the Tibetan Children’s Village, to serve as population references. Genotype data from 573 persons of Jewish descent who represent 16 populations, including two Sephardi populations, were collected from the Jewish Hapmap Project [7] and served as a Jewish ancestry reference group. Single nucleotide polymorphism (SNP) genotypes from 471 previously studied individuals of Ashkenazi Jewish descent [8] were also analyzed. 423 HapMap samples (62 CEPH (Utah residents with ancestry from northern and western Europe; CEU); 45 Han Chinese in Beijing, China (CHB); 90 Chinese in metropolitan Denver, Colorado (CHD); 90 Gujarati Indians in Houston, Texas (GIH); 46 Japanese in Tokyo, Japan (JPT); 90 Toscani in Italy (TSI)) were included in the analysis to provide a broader assessment of Eurasian population genetic structure. Previously collected genotypes from 25 Buryats [9], 24 Kurds [9], 25 Kyrgyzstanis [9], 94 Tibetans [10, 11], 42 Mongolians (Qinghai) [12], and 26 Slovenians [9] were also analyzed. In total Affymetrix SNP 6.0 array data were assembled from 1,750 individuals (Table 1).

**Table 1:** A summary of analyzed samples and how many were removed due to quality control measures.

Source	Population	Number of samples before QC	Samples removed by contrast QC	Samples with low (<95%) genotyping rate	Related samples removed	Samples removed by PCA	Total samples used for analysis
**Samples gathered in Kashmir Valley**							
	Kashmiri*^	15	0	3	0	0	12
	Kashmiri Tibetan*^	16	0	5	4	0	7
**Samples gathered in McLeod Ganj, India**							
	Tibetan*^	14	0	3	0	0	11
	Tibetan Children’s Village*^	2	0	0	0	0	2
**HapMap Project**							
	CEU*	62	0	3	0	0	59
	TSI*	90	0	3	0	0	87
	GIH*	90	2	5	4	0	79
	CHD*	90	0	6	2	0	82
	CHB*	45	0	1	0	0	44
	JPT*	46	0	0	0	0	46
**Jewish HapMap Project**							
	Algerian*	24	1	0	0	0	23
	Ashkenazi*	36	0	13	0	0	23
	Djerbian*	18	0	2	7	6	3
	Georgian*	15	1	1	0	0	13
	Indian (Cochin)*	20	0	0	5	0	15
	Indian (random)*	20	0	0	1	0	19
	Iranian*	119	28	82	1	0	8
	Iraqi*	40	1	2	16	0	21
	Italian*	28	0	4	8	0	16
	Libyan*	38	1	1	0	21	15
	Moroccan*	38	1	1	0	0	36
	Sephardi (Greece)*	60	0	0	17	0	43
	Sephardi (Turkey)*	19	0	2	1	0	16
	Syrian*	33	0	0	11	0	22
	Tunisian*	29	0	0	0	7	22
	Yemeni*	36	3	1	0	4	28
Bray et al., 2010*							
	Ashkenazi Jewish*	471	0	3	0	0	468
**In-house individuals previously collected**							
	Tibetan—Maduo*	31	0	0	0	0	31
	Tibetan—Tuo Tuo River*	46	0	0	0	0	46
	Tibetan—Refugees in Utah*	17	0	0	2	0	15
	Slovenian*	26	0	3	1	0	22
	Kurdish*	24	0	0	0	0	24
	Buryatan*	25	0	2	0	9	14
	Kyrgyzstani*	25	0	0	0	1	24
	Mongolian (Qinghai)*	42	0	1	0	0	41
**Human Genome Diversity Project (Lopez Herraez et al., 2009)**							
	Adygei	5	NA	NA	0	0	5
	Balochi	5	NA	NA	0	0	5
	Basque	5	NA	NA	0	0	5
	Bedouin	5	NA	NA	0	0	5
	Bergamo	5	NA	NA	0	0	5
	Brahui	5	NA	NA	0	0	5
	Burusho	5	NA	NA	0	0	5
	Cambodian	5	NA	NA	0	0	5
	Dai	5	NA	NA	1	0	4
	Daur	5	NA	NA	0	0	5
	Druze	5	NA	NA	0	0	5
	French	5	NA	NA	0	0	5
	Han	5	NA	NA	0	0	5
	Hazara	5	NA	NA	0	0	5
	Hezhen	5	NA	NA	1	0	4
	Japanese	5	NA	NA	0	0	5
	Kalash	5	NA	NA	1	0	4
	Lahu	5	NA	NA	2	0	3
	Miaozu	5	NA	NA	0	0	5
	Mongola	5	NA	NA	0	0	5
	Naxi	5	NA	NA	0	0	5
	Orcadian	5	NA	NA	1	0	4
	Oroqen	5	NA	NA	0	1	4
	Palestinian	5	NA	NA	0	0	5
	Pathan	5	NA	NA	0	0	5
	Russian	5	NA	NA	0	0	5
	Sardinian	5	NA	NA	0	0	5
	She	5	NA	NA	2	0	3
	Sindhi	5	NA	NA	0	0	5
	Tu	5	NA	NA	0	0	5
	Tujia	5	NA	NA	0	0	5
	Tuscan	5	NA	NA	0	0	5
	Uygur	5	NA	NA	0	0	5
	Xibo	5	NA	NA	0	0	5
	Yakut	5	NA	NA	0	5	0
	Yizu	5	NA	NA	0	0	5
**Indian subcontinent (Moorjani et al., 2013)**							
	Adi-Dravider	5	NA	NA	0	0	5
	Aonaga	4	NA	NA	1	0	3
	Bhil	17	NA	NA	0	0	17
	Bhumij	5	NA	NA	0	0	5
	Birhor	4	NA	NA	0	0	4
	Brahmin	15	NA	NA	7	0	8
	Changapa	5	NA	NA	0	0	5
	Chenchu	6	NA	NA	5	0	1
	Gond	14	NA	NA	0	0	14
	Gounder	5	NA	NA	0	0	5
	Hallaki	7	NA	NA	0	0	7
	Ho	5	NA	NA	0	0	5
	Irula	5	NA	NA	0	0	5
	Jains	5	NA	NA	0	0	5
	Jewish (Indian)	5	NA	NA	0	0	5
	Kallar	5	NA	NA	0	0	5
	Kamsali	4	NA	NA	0	0	4
	Kashmiri Pandit	20	NA	NA	5	0	15
	Kattunayakkan	5	NA	NA	0	0	5
	Kharia	6	NA	NA	0	0	6
	Korku	4	NA	NA	1	0	3
	Kshatriya	20	NA	NA	6	0	14
	Kuruchiyan	5	NA	NA	0	0	5
	Kurumba	9	NA	NA	0	0	9
	Lodi	5	NA	NA	0	0	5
	Madiga	19	NA	NA	7	0	12
	Mala	18	NA	NA	6	0	12
	Malai Kuravar	5	NA	NA	0	0	5
	Malli	5	NA	NA	0	0	5
	Meghawal	5	NA	NA	0	0	5
	Minicoy	5	NA	NA	0	0	5
	Munda	5	NA	NA	0	0	5
	Naidu	4	NA	NA	0	0	4
	Narikkuravar	5	NA	NA	2	0	3
	Nysha	4	NA	NA	2	0	2
	Palliyar	5	NA	NA	0	0	5
	Paniyas	5	NA	NA	3	0	2
	Sahariya	4	NA	NA	0	0	4
	Santhal	7	NA	NA	0	0	7
	Satnami	4	NA	NA	0	0	4
	Sherpa	5	NA	NA	3	0	2
	Srivastava	2	NA	NA	0	0	2
	Subba	5	NA	NA	1	0	4
	Tharu	9	NA	NA	0	0	9
	Tibet refugees	5	NA	NA	0	0	5
	Vaish	4	NA	NA	0	0	4
	Vedda	4	NA	NA	2	0	2
	Velama	4	NA	NA	0	0	4
	Vysya	20	NA	NA	6	0	14
**European continental populations (Lao et al., 2008)**							
	Austria	50	NA	NA	0	0	50
	Czech Republic	45	NA	NA	0	0	45
	Denmark	59	NA	NA	0	0	59
	Finland	47	NA	NA	0	0	47
	France	50	NA	NA	0	0	50
	Germany	983	NA	NA	0	0	983
	Hungary	17	NA	NA	0	0	17
	Ireland	35	NA	NA	0	0	35
	Italy	155	NA	NA	0	0	155
	Netherlands	280	NA	NA	0	0	280
	Northern Greece	51	NA	NA	0	0	51
	Norway	52	NA	NA	0	0	52
	Poland	49	NA	NA	0	0	49
	Portugal	16	NA	NA	0	0	16
	Romania	12	NA	NA	0	0	12
	Spain	128	NA	NA	0	0	128
	Sweden	46	NA	NA	0	0	46
	Switzerland	133	NA	NA	0	0	133
	UK	194	NA	NA	0	0	194
	Former Yugoslavia	55	NA	NA	0	0	55
	**Dataset total**	4735	38	147	145	54	4,351

* = genotyped on the Affymetrix SNP 6.0 genotyping array.

^ = DNA samples collected and genotyped in this study.

Contrast quality-control (QC) measurements, which measure how well homozygote and heterozygote genotype calls can be differentiated on the genotyping chip, were performed on each sample array using the Affymetrix Genotyping Console Software. Sample arrays that had poor contrast QC measurement (>0.40) were removed from the analysis. For arrays that passed QC, genotypes were called using the Birdseed-v2 algorithm [13] in the Affymetrix Genotyping Console Software. Only autosomal SNP genotypes were retained for analysis. Samples that had low outlier call rates (<95% genotyping rate) were removed from further analysis. SNPs that had a minor allele frequency less than 0.05 and a genotyping call rate less than 0.05 were filtered from the dataset. The method of moments identity by descent (IBD) procedure in the PLINK software package [14, 15] was used to identify pairs of individuals related at the first cousin level or closer; one member of each pair was removed from analysis.

Additional samples that were not assayed on the Affymetrix 6.0 array were collected in order to assess finer genetic structure in Eurasian populations, specifically that of Greeks and samples from the Indian subcontinent. 180 samples, which were SNP genotyped on the Affymetrix GeneChip Human Mapping 500 K Array Set, representing 36 populations were selected from the Human Genome Diversity Project [16], were included. Genotypes in this dataset were converted from hg18 to hg19 genomic coordinates using the UCSC liftOver tool [17]; unmappable SNPs were removed. 348 previously studied individuals from 49 populations in the Indian subcontinent [18] were used to better compare the Kashmiri population with surrounding populations. Lastly, a large number of previously studied individuals (2,457) from 20 European countries [19], including Greek and other Balkan populations, were included to test whether the Kashmiri population contains Greek admixture. SNPs that had a minor allele frequency less than 0.05 and a genotyping call rate less than 0.05 were omitted from each respective dataset. The three aforementioned datasets were merged with the Affymetrix SNP 6.0 dataset after removing ambiguous SNPs to avoid strand orientation errors. Genotype filtering and dataset merging were performed using the PLINK [14] and PLINK 1.9 [20] software packages and custom scripts.

Several methods were used to test for evidence of global Greek or Jewish ancestry in the Kashmiri population and to determine the ancestry of Tibetans from Mcleod Ganj. First, a principal components analysis (PCA) was performed on the SNP genotype dataset to determine whether genetic outliers existed and to identify inter-individual genetic similarity. PCA was performed using the *Smartpca* program in the *EIGENSOFT* package version 6.0.1 [21, 22]. Samples that appeared to be extreme outliers (likely due to genotyping error or strong genetic dissimilarity from the rest of the dataset) were removed. Extreme genetic outliers were defined as samples that were 6.0 standard deviations away from the mean principal component value for the first 10 principal components. F_st_ values, which measure population differentiation, were also calculated (*Smartpca*) in a pairwise fashion to determine the amount of differentiation between each population. Next, individual ancestry estimation was performed using the *ADMIXTURE* software tool [23]. *ADMIXTURE* is a model-based global ancestry estimation approach that estimates the proportion of ancestry derived from K ancestral populations for each studied individual from genotype data. In order to control for linkage disequilibrium (LD), which can skew *ADMIXTURE* results, markers found to be in LD were removed from the dataset specifically for *ADMIXTURE* analysis using the PLINK 1.9 ‘indep-pairwise’ parameter [20]. A sliding window of 50 SNPs was used, removing SNP pairs with an r^2^ value above 0.1. *ADMIXTURE* was performed assuming the number of ancestral populations ranged from 1 to 10. The cross-validation error was recorded for each *ADMIXTURE* test to determine the most likely model. Lastly, the f_3_ test [24] was utilized to specifically determine whether the Kashmiri population derives a significant amount of ancestry from two selected ancestral populations, thus indicating an admixture event. Every pairwise combination (8,001 total) of the populations in this study (excluding the Kashmiri population) was used as the two ancestral populations in the f_3_ test. Any f_3_ test with a negative score and a significant z-score (z < -1.64) was considered to be evidence of admixture in the Kashmiri population arising from the two selected ‘ancestral’ populations.

## Results

In total, 38 sample arrays failed the contrast QC step and were removed from the analysis (Table 1), and an additional 147 samples (including 3 Kashmiri samples) were removed due to low (<95%) genotyping rates (Table 1). 145 subjects were removed because they were related at the first cousin (or closer) level to another study subject (Table 1). 93,666 autosomal SNP genotypes were retained for these samples after genotype filtering and merging each dataset into one unified PLINK file. PCA identified 54 samples as genetic outliers that were subsequently discarded from further consideration (Table 1). 1,437 samples that were genotyped by the Affymetrix SNP 6.0 array passed all QC standards. 4,351 samples in total, including 12 Kashmiris, were used for analysis.

**Fig 1:**
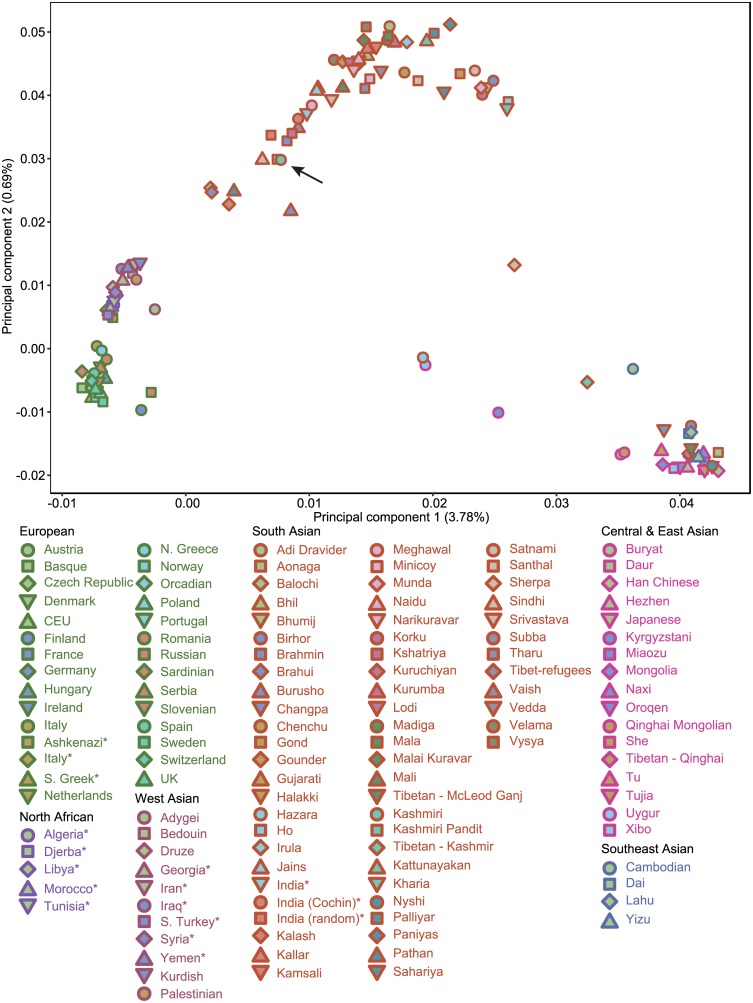
A principal components plot of principal components 1 and 2 representing the studied genotypic data. Each population is plotted according to the mean principal component value across all individuals belonging to the respective population. The black arrow shows where the Kashmiri samples cluster. The color of the outline of each symbol corresponds to the broader population group. * indicates Jewish populations. The abbreviation S. stands for Sephardic.

The PCA demonstrates clear genetic patterning of populations in European, South Asian, and East Asian subcontinental groups (first two principal components, shown in Fig 1). A number of populations fall between the subcontinental genetic clusters (North African, West Asian, South Asian, and some East Asian populations). The Kashmiri samples are grouped near other previously studied groups from northern India and Pakistan, which indicates similar genetic ancestry. Further, the mean principal component 1 and 2 coordinate of a previously studied population in Kashmir (15 Kashmiri Pandits) is found to plot in the center of the collected Kashmiri cluster, indicating similar heritage and validating the quality of the Kashmiri genotypes. A number of populations residing in nearby Pakistan also show genetic similarity to the Kashmiris, including the Burusho, Balochi, Brahui, Sindhi, and Kalash. Principal components 1 and 2 also show that the Indo-European ethno-linguistic populations from northern India lie on a cline between western European populations and Dravidian ethno-linguistic groups of southern India. This pattern suggests that Indo-European ethno-linguistic groups of northern India, including the Kashmiris, share a complex ancestral history with both west Eurasian and Indian populations. None of the Kashmiris clustered near the northern Greek or Sephardic Jewish (Greece and Turkey) populations in principal components 1 and 2. A similar pattern is observed in principal components 3 and 4 (Fig A in S1 Appendix).

The PCA also showed evidence of genetic heterogeneity and admixture in the Kashmiri Tibetan samples. The Kashmiri Tibetans plotted more closely to the populations of South Asia than to the Tibetan reference populations that include Tibetans from Mcleod Ganj (Fig 1). Furthermore, the standard deviation of the Kashmiri Tibetans’ first component is much higher (0.0048) than that of other Tibetans (0.00078), which includes Tibetans in Mcleod Ganj and Qinghai. This pattern is suggestive of recent Tibetan admixture with populations from northern India or west Asia.

F_st_ estimates also show that the Kashmiris are very genetically similar to the 15 previously studied Kashmiri Pandits, as expected if the Kashmiri samples represent individuals from the region (Table A in S1 Appendix). F_st_ analysis also shows that the Kashmiris are very genetically similar to other nearby South Asian Indo-European linguistic populations in northern India and Pakistan; the 10 closest related populations are of northern Indian or Pakistani origin (Table A in S1 Appendix). Larger genetic distances with these nearby populations would be expected if Kashmiris had substantial amounts of Greek or Sephardic Jewish ancestry. The northern Greek population had a genetic distance of 0.021 with the Kashmiri population, while the Turkish Sephardic Jews and Greek Sephardic Jews had distances of 0.021 and 0.022, respectively. These genetic distances are approximately the same as those between persons from Italy and persons of Finnish descent [25]. The mean genetic distance of European populations with the Kashmiris was 0.024. European similarity seen in Kashmiris does not reflect a contribution from a specific European population because a majority of the European F_st_ values are near this mean value.

**Fig 2:**
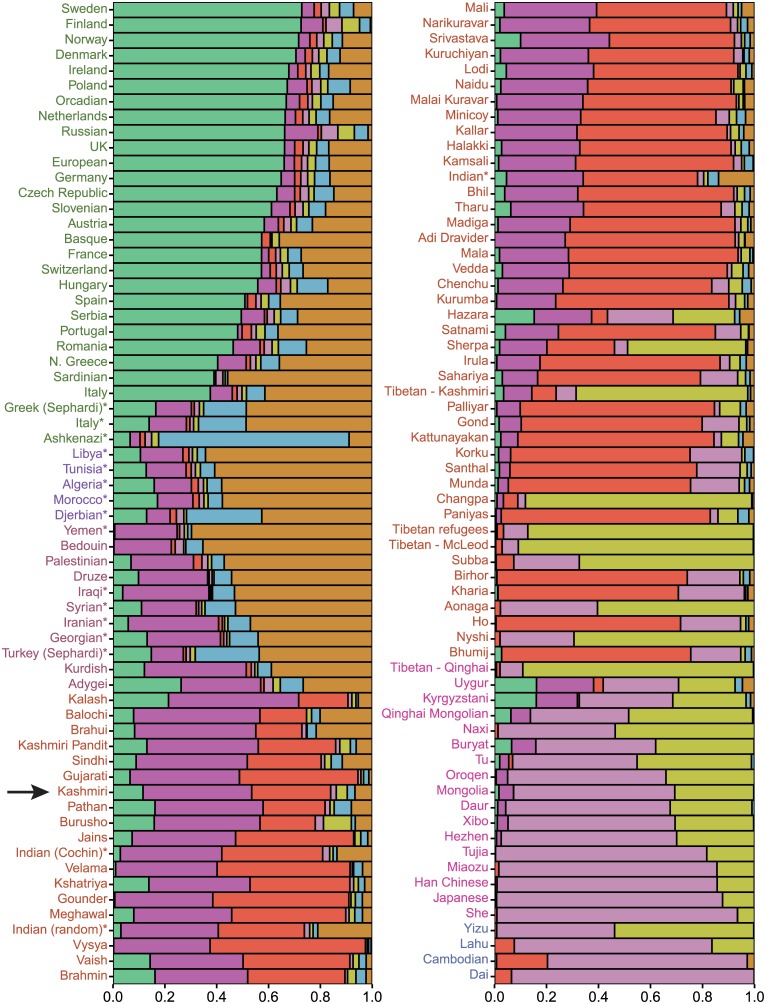
An *ADMIXTURE* plot showing the proportion of ancestry each hypothetical ancestral population (K = 7) contributes to each studied population. The mean admixture proportion of each component across each given population was calculated and the sum rescaled to one. The black arrow indicates the admixture proportions of the Kashmiri population. * indicates Jewish populations. The admixture profile of the Kashmiri population is similar to other northern Indian and Pakistani populations.

While PCA and F_st_ can robustly determine how genetically similar distinct populations or individuals are to each other, they do not directly estimate individual ancestry proportions. Model-based approaches, such as *ADMIXTURE* [23], attempt to estimate the relative contributions of each of K ancestral populations to each individual’s genetic makeup. A K value of 7 had the smallest level of cross-validation error (cross-validation error = 0.56024; Fig D in S1 Appendix), indicating it is the most likely ancestral population model. However, the small difference in cross-validation error ranging from K = 6 to K = 10 implies that other models could potentially explain the data as well. While *ADMIXTURE* ancestry proportions should not be considered direct estimates of admixture levels [26], the overall pattern of ancestry sharing can be used to determine if populations are genetically similar. The *ADMIXTURE* profile of Kashmiris shows a high degree of similarity with other geographically nearby populations. Specifically, the Kashmiris in the K = 7 (Fig 2) *ADMIXTURE* model possess ancestry profiles similar to those of Indo-European and Dravidian ethno-linguistic populations in India and Pakistan. Further, none of the ancestral components of the Kashmiris appear to be derived specifically from Greeks or Sephardic Jews, which would make the Kashmiris appear dissimilar from nearby populations. The overall *ADMIXUTRE* profiles of these Indian and Pakistani populations are distinct from those of west Eurasian populations and especially those of East Asian descent. These general patterns largely hold true for K = 5 through K = 10 (Figs H-L in S1 Appendix). Of note, the ancestry profile found in the Kashmiris is virtually indistinguishable from the 15 previously studied Kashmiri Pandit samples. Interestingly, the ancestry profile of the Kashmiri Tibetans is somewhat dissimilar from those found in Tibetan reference populations as it possesses ancestry components found largely in west Eurasian and South Asian populations.

**Fig 3:**
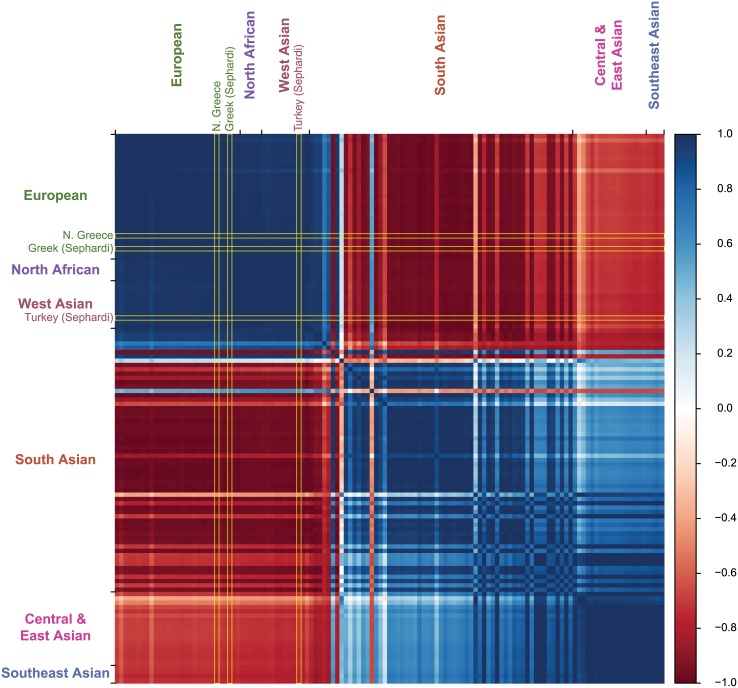
A heatmap displaying the amount that the f_3_ results of a particular population are correlated with another population. The intersection of a row and column represents how correlated the f_3_ results are of those two populations. The columns and rows are in the same order as displayed in Fig 2. Darker shades of blue indicate that the two populations in question have strongly correlated f_3_ results whereas darker shades of red indicate anti-correlation. The geographical groupings of the subpopulations are indicated in the margins of the Fig The correlation comparisons of the northern Greek, Sephardic Greek, and Sephardic Turkish populations are highlighted in yellow. The f_3_ results of west Eurasian populations are highly correlated with each other. South Asian populations also tend to be correlated with other populations of South Asian ancestry.

While model-based analysis provides direct estimates of ancestry proportions, it is not a formal statistical test of admixture. The f_3_ statistic [24] provides a formal statistical test of the hypothesis that a pair of ‘ancestral’ populations contributed to the ancestry of a present-day population. 3,366 of 8,001 total pairs showed evidence of jointly contributing to the Kashmiri population. 69 of the significant pairwise tests involved the northern Greek population (Table 2). These 69 significant tests involved populations from South Asia (50), Central and East Asia (17), (Table 2). The results could suggest that an admixture event took place between Greeks and an ancestral South or East Asian population, contributing to the Kashmiri population. However, European and west Eurasian populations show almost identical f_3_ results with the Northern Greek population. For instance, the z-scores of the f_3_ tests that involve the northern Greek population are nearly perfectly correlated with those of populations of European, North African, and West Asian ancestry (r = 0.994 on average) (Fig 3). The Sephardic Jewish populations from Greece and Turkey both have nearly identical f_3_ results that were as suggestive of Kashmiri admixture as the northern Greeks (Table 2). The f_3_ results of both Sephardic Jewish populations are also highly correlated with those of European and West Asian populations, just as the northern Greeks. These results together suggest that Kashmiris generally share ancestry with west Eurasian and South Asian populations, as opposed to having directly received significant genetic contributions from Greek or Jewish populations. The full list of f_3_ results can be found in S1 Dataset.

**Table 2:** A summary of the number of significant f_3_ tests per population.

Population group	Population	European (29)	North African (5)	West Asian (11)	South Asian (61)	Central & East Asian (17)	Southeast Asian (4)	Total (127)
European								
	Austria	0	0	0	50	15	4	69
	Basque	0	0	0	49	15	4	68
	Czech Republic	0	0	0	50	15	4	69
	Denmark	0	0	0	48	15	4	67
	European	0	0	0	50	15	4	69
	Finland	0	0	0	48	7	4	59
	France	0	0	0	50	15	4	69
	Germany	0	0	0	50	15	4	69
	Hungary	0	0	0	50	15	4	69
	Ireland	0	0	0	48	15	4	67
	Italy	0	0	0	50	15	4	69
	Ashkenazi*	0	0	0	49	15	4	68
	Italy*	0	0	0	49	15	4	68
	Greek (Sephardi)*	0	0	0	49	15	4	68
	Netherlands	0	0	0	50	15	4	69
	Northern Greece	0	0	0	50	15	4	69
	Norway	0	0	0	49	15	4	68
	Orcadian	0	0	0	48	15	4	67
	Poland	0	0	0	50	15	4	69
	Portugal	0	0	0	49	15	4	68
	Romania	0	0	0	50	15	4	69
	Russian	0	0	0	46	6	4	56
	Sardinian	0	0	0	49	15	4	68
	Serbia	0	0	0	50	15	4	69
	Slovenian	0	0	0	50	15	4	69
	Spain	0	0	0	50	15	4	69
	Sweden	0	0	0	48	15	4	67
	Switzerland	0	0	0	50	15	4	69
	UK	0	0	0	50	15	4	69
North African								
	Algeria*	0	0	0	50	16	4	70
	Djerbian*	0	0	0	49	15	4	68
	Libya*	0	0	0	49	15	4	68
	Morocco*	0	0	0	49	15	4	68
	Tunisia*	0	0	0	49	15	4	68
West Asian								
	Adygei	0	0	0	50	15	4	69
	Bedouin	0	0	0	49	15	4	68
	Druze	0	0	0	50	16	4	70
	Georgian*	0	0	0	50	16	4	70
	Iranian*	0	0	0	51	16	4	71
	Iraqi*	0	0	0	50	16	4	70
	Turkey (Sephardi)*	0	0	0	49	15	4	68
	Syrian*	0	0	0	50	16	4	70
	Yemen*	0	0	0	49	16	4	69
	Kurdish	0	0	0	51	16	4	71
	Palestinian	0	0	0	49	15	4	68
South Asian								
	Adi Dravider	29	5	11	5	0	0	50
	Aonaga	29	5	11	4	0	0	49
	Balochi	0	0	0	42	16	4	62
	Bhil	29	5	11	5	0	0	50
	Bhumij	29	5	11	4	0	0	49
	Birhor	29	5	11	4	0	0	49
	Brahmin	0	1	7	0	0	0	8
	Brahui	0	0	0	45	16	4	65
	Burusho	0	0	0	17	0	0	17
	Changpa	29	5	11	5	0	0	50
	Chenchu	29	5	11	4	0	0	49
	Gond	29	5	11	4	0	0	49
	Gounder	29	5	11	3	0	0	48
	Gujarati	29	5	11	4	0	0	49
	Halakki	29	5	11	4	0	0	49
	Hazara	0	0	0	0	0	0	0
	Ho	29	5	11	4	0	0	49
	Irula	29	5	11	4	0	0	49
	Jains	29	5	11	2	0	0	47
	Indian*	24	0	3	2	0	0	29
	Indian (Cochin)*	0	0	0	0	0	0	0
	Indian (random)*	0	0	0	0	0	0	0
	Kalash	0	0	0	46	16	4	66
	Kallar	29	5	11	5	0	0	50
	Kamsali	29	5	11	4	0	0	49
	Kashmiri Pandit	0	0	0	6	8	1	15
	Tibetan (Kashmiri)	28	5	11	5	0	0	49
	Kattunayakan	29	5	11	5	0	0	50
	Kharia	29	5	11	5	0	0	50
	Korku	29	5	11	5	0	0	50
	Kshatriya	21	5	11	1	0	0	38
	Kuruchiyan	29	5	11	4	0	0	49
	Kurumba	29	5	11	5	0	0	50
	Lodi	29	5	11	4	0	0	49
	Madiga	29	5	11	5	0	0	50
	Mala	29	5	11	5	0	0	50
	Malai Kuravar	29	5	11	5	0	0	50
	Mali	29	5	11	2	0	0	47
	Tibetan (McLeod Ganj)	29	5	11	6	0	0	51
	Meghawal	29	5	11	0	0	0	45
	Minicoy	29	5	11	4	0	0	49
	Munda	29	5	11	5	0	0	50
	Naidu	29	5	11	2	0	0	47
	Narikuravar	29	5	11	4	0	0	49
	Nyshi	29	5	11	4	0	0	49
	Palliyar	29	5	11	4	0	0	49
	Paniyas	29	5	11	6	0	0	51
	Pathan	0	0	0	37	15	4	56
	Sahariya	29	5	11	4	0	0	49
	Santhal	29	5	11	4	0	0	49
	Satnami	29	5	11	4	0	0	49
	Sherpa	29	5	11	5	0	0	50
	Sindhi	0	0	0	5	10	1	16
	Srivastava	29	5	11	4	0	0	49
	Subba	29	5	11	6	0	0	51
	Tharu	29	5	11	5	0	0	50
	Tibet-refugees	28	5	11	4	0	0	48
	Vaish	23	5	10	0	0	0	38
	Vedda	29	5	11	3	0	0	48
	Velama	29	5	11	1	0	0	46
	Vysya	29	5	11	5	0	0	50
Central & East Asian								
	Buryat	27	5	11	5	0	0	48
	Daur	27	5	11	6	0	0	49
	Han Chinese	29	5	11	6	0	0	51
	Hezhen	27	5	11	6	0	0	49
	Japanese	29	5	11	5	0	0	50
	Kyrgyzstani	0	1	7	3	0	0	11
	Miaozu	29	5	11	6	0	0	51
	Mongolia	27	5	11	4	0	0	47
	Naxi	29	5	11	6	0	0	51
	Oroqen	27	5	11	4	0	0	47
	Qinghai Mongolian	27	5	11	6	0	0	49
	She	29	5	11	4	0	0	49
	Tibetan (Qinghai)	28	5	11	5	0	0	49
	Tu	27	5	11	5	0	0	48
	Tujia	29	5	11	6	0	0	51
	Uygur	0	0	0	0	0	0	0
	Xibo	27	5	11	4	0	0	47
Southeast Asian								
	Cambodian	29	5	11	4	0	0	49
	Dai	29	5	11	5	0	0	50
	Lahu	29	5	11	5	0	0	50
	Yizu	29	5	11	4	0	0	49

Significant f_3_ tests were classified as those that had a negative score and z-score < -1.64. Each column contains the group in which the other tested ancestral population is found. The continental groupings for each column follow the same scheme used in the left hand side of this table. Each number in the column heading is the total number of populations within each continental group.

* indicates Jewish populations.

## Discussion

Our results do not support the hypothesis of a substantial genome-wide Greek or Sephardic ancestral contribution to the Kashmiri population. This finding is consistent with previous literature that found no evidence of Jewish admixture in Kashmiris using Y chromosome haplotype data [27]. In addition, we found no evidence of substantial Greek admixture in the Burusho, Kalash, or Pathans, which have all been suggested to have ancestry derived from Alexander the Great’s armies and the Greeks [6, 27–30]. The PCA and *ADMIXTURE* results suggest that the Kashmiris are very similar genetically to other geographically proximate populations. The f_3_ results suggest that the Kashmiris derive a significant amount of ancestry from Indian/South Asian and western Eurasian sources. These results, together with those of previous investigations, suggest that substantial Greek or Jewish admixture did not occur specifically in the Kashmiri population. Instead, the results suggest that the Kashmiri population, and nearby surrounding populations, share genetic ancestry broadly with west Eurasian and South Asian populations.

There are, however, a number of possible reasons why recent Greek or Jewish admixture might be undetected in these analyses. It is possible that more cryptic admixture, in the form of specific Greek or Jewish autosomal haplotypes, exists. Tests such as *rolloff* [24], *ALDER* [31], and *GLOBETROTTER* [32] can detect admixture by utilizing linkage disequilibrium and haplotype data. However, this study did not have sufficient SNP density (93,666 autosomal SNPs) to capture linkage disequilibrium and haplotype structure. High-density genotyping array or next-generation whole genome sequencing, applied widely in diverse populations, would provide these data.

Another potential explanation for the lack of Greek and Jewish ancestry in the Kashmiris is that the Kashmiris sampled here are not representative of those who lived when the supposed admixture event took place more than 2,000 years ago. The same is true of the putative Greek and Jewish ancestral populations. As previously discussed, there is archeological evidence to suggest that the ancient Greeks were in the Kashmir region [3]. Another limitation of this study is the small Kashmiri sample size. It is reassuring, however, that this sample is genetically very similar to the 15 previously studied Kashmiri Pandits.

It is also possible that the Southern European and Mediterranean admixture seen in the Kashmiri individuals represents Greek or Sephardic Jewish ancestry. However, these patterns are not Kashmiri-specific and are seen in a number of nearby Indo-European ethno-linguistic populations in northern India and Pakistan. Taken together, these findings suggest strongly that the Kashmiri population is genetically similar to nearby populations and does not have a distinctly different ancestral origin.

Lastly, we noted that the Kashmiri Tibetans displayed ancestry from both Tibetan and South/West Asian sources. The Kashmiri Tibetans show ancestry deriving from the various populations of India, Pakistan, and western Asia (Fig 2). The degree of ancestry deriving from these populations in the Kashmiri Tibetans is also highly variable, which is a pattern consistent with recent admixture. Ancestry from these populations could suggest that admixture took place between Tibetans and Arabic-speaking peoples in the past. Such events are thought to have occurred as early as the eighth century A.D. when Islam was first introduced to Tibet [33]. In addition, some Kashmiri Tibetans claim to have originated from Kashmir, migrated to Lhasa, and returned to India after the incorporation of Tibet into the People's Republic of China. These migratory events could have resulted in additional admixture.

Our results also show that the Tibetans in McLeod Ganj are very genetically similar to previously studied Tibetan populations found on the Tibetan Plateau. As a result, studies of this population could be useful in elucidating the genetic and physiological mechanisms by which Tibetans are able to adapt and survive in high altitude and hypoxic conditions.

## Supporting Information

S1 Appendix Supplementary Appendix. **Supporting Figures**: Fig A) A principal components plot of principal components 3 and 4 representing the studied genotypic data. Fig B) A principal components plot of principal components 1 and 2 representing the studied genotypic data including 60 unrelated Yoruban individuals genotyped on the Affymetrix SNP 6.0 array. Fig C) A principal components plot of principal components 3 and 4 representing the studied genotypic data including 60 unrelated Yoruban individuals genotyped on the Affymetrix SNP 6.0 array. Fig D) A plot of the cross-validation error vs. varying levels of K in the *ADMIXTURE* analysis. Fig E) An *ADMIXTURE* plot showing the proportion of ancestry each hypothetical ancestral population (K = 2) contributes to each studied population. Fig F) An *ADMIXTURE* plot showing the proportion of ancestry each hypothetical ancestral population (K = 3) contributes to each studied population. Fig G) An *ADMIXTURE* plot showing the proportion of ancestry each hypothetical ancestral population (K = 4) contributes to each studied population. Fig H) An *ADMIXTURE* plot showing the proportion of ancestry each hypothetical ancestral population (K = 5) contributes to each studied population. Fig I) An *ADMIXTURE* plot showing the proportion of ancestry each hypothetical ancestral population (K = 6) contributes to each studied population. Fig J) An *ADMIXTURE* plot showing the proportion of ancestry each hypothetical ancestral population (K = 8) contributes to each studied population. Fig K) An *ADMIXTURE* plot showing the proportion of ancestry each hypothetical ancestral population (K = 9) contributes to each studied population. Fig L) An *ADMIXTURE* plot showing the proportion of ancestry each hypothetical ancestral population (K = 10) contributes to each studied population. **Supporting Tables. Table A)** F_st_ genetic distance values relative to the Kashmiri population in ascending order.
(DOCX)

S1 Dataset A complete list of the f_3_ results. (TXT)
