# Supplementary material for: A Genome-Wide Search for Greek and Jewish Admixture in the Kashmiri Population
Source: PLoS One. 2016 Aug 4;11(8):e0160614. doi: 10.1371/journal.pone.0160614 (PMC4973929; doi:10.1371/journal.pone.0160614)
Supplement: S1 Appendix — Supporting Figures: Fig A) A principal components plot of principal components 3 and 4 representing the studied genotypic data. Fig B) A principal components plot of principal components 1 and 2 representing the studied genotypic data including 60 unrelated Yoruban individuals genotyped on the Affymetrix SNP 6.0 array. Fig C) A principal components plot of principal components 3 and 4 representing the studied genotypic data including 60 unrelated Yoruban individuals genotyped on the Affymetrix SNP 6.0 array. Fig D) A plot of the cross-validation error vs. varying levels of K in the ADMIXTURE analysis. Fig E) An ADMIXTURE plot showing the proportion of ancestry each hypothetical ancestral population (K = 2) contributes to each studied population. Fig F) An ADMIXTURE plot showing the proportion of ancestry each hypothetical ancestral population (K = 3) contributes to each studied population. Fig G) An ADMIXTURE plot showing the proportion of ancestry each hypothetical ancestral population (K = 4) contributes to each studied population. Fig H) An ADMIXTURE plot showing the proportion of ancestry each hypothetical ancestral population (K = 5) contributes to each studied population. Fig I) An ADMIXTURE plot showing the proportion of ancestry each hypothetical ancestral population (K = 6) contributes to each studied population. Fig J) An ADMIXTURE plot showing the proportion of ancestry each hypothetical ancestral population (K = 8) contributes to each studied population. Fig K) An ADMIXTURE plot showing the proportion of ancestry each hypothetical ancestral population (K = 9) contributes to each studied population. Fig L) An ADMIXTURE plot showing the proportion of ancestry each hypothetical ancestral population (K = 10) contributes to each studied population. Supporting Tables. Table A) Fst genetic distance values relative to the Kashmiri population in ascending order. (DOCX) [file pone.0160614.s001.docx]

**Supplementary Appendix**

**TABLE OF CONTENTS**

1. **Supporting Figures**
   1. A principal components plot of principal components 3 and 4 representing the studied genotypic data.
   2. A principal components plot of principal components 1 and 2 representing the studied genotypic data including 60 unrelated Yoruban individuals genotyped on the Affymetrix SNP 6.0 array.
   3. A principal components plot of principal components 3 and 4 representing the studied genotypic data including 60 unrelated Yoruban individuals genotyped on the Affymetrix SNP 6.0 array.
   4. A plot of the cross-validation error vs. varying levels of K in the *ADMIXTURE* analysis.
   5. An *ADMIXTURE* plot showing the proportion of ancestry each hypothetical ancestral population (K = 2) contributes to each studied population.
   6. An *ADMIXTURE* plot showing the proportion of ancestry each hypothetical ancestral population (K = 3) contributes to each studied population.
   7. An *ADMIXTURE* plot showing the proportion of ancestry each hypothetical ancestral population (K = 4) contributes to each studied population.
   8. An *ADMIXTURE* plot showing the proportion of ancestry each hypothetical ancestral population (K = 5) contributes to each studied population.
   9. An *ADMIXTURE* plot showing the proportion of ancestry each hypothetical ancestral population (K = 6) contributes to each studied population.
   10. An *ADMIXTURE* plot showing the proportion of ancestry each hypothetical ancestral population (K = 8) contributes to each studied population.
   11. An *ADMIXTURE* plot showing the proportion of ancestry each hypothetical ancestral population (K = 9) contributes to each studied population.
   12. An *ADMIXTURE* plot showing the proportion of ancestry each hypothetical ancestral population (K = 10) contributes to each studied population.
2. **Supporting Tables**
3. F_st_ genetic distance values relative to the Kashmiri population in ascending order.

**I. Supporting Figures**


**Figure A. A principal components plot of principal components 3 and 4 representing the studied genotypic data.** Each population is plotted according to the mean principal component value across all individuals belonging to the respective population. The black arrow shows where the Kashmiri samples cluster. The color of the outline of each symbol corresponds to the broader population group. The abbreviation S. stands for Sephardic.

**Figure B. A principal components plot of principal components 1 and 2 representing the studied genotypic data including 60 unrelated Yoruban individuals genotyped on the Affymetrix SNP 6.0 array.** Each population is plotted according to the mean principal component value across all individuals belonging to the respective population. The black arrow shows where the Kashmiri samples cluster. The color of the outline of each symbol corresponds to the broader population group. The abbreviation S. stands for Sephardic.

**Figure C. A principal components plot of principal components 3 and 4 representing the studied genotypic data including 60 unrelated Yoruban individuals genotyped on the Affymetrix SNP 6.0 array.**  Each population is plotted according to the mean principal component value across all individuals belonging to the respective population. The black arrow shows where the Kashmiri samples cluster. The color of the outline of each symbol corresponds to the broader population group. The abbreviation S. stands for Sephardic.

**Figure D. A plot of the cross-validation error vs. varying levels of K in the *ADMIXTURE* analysis.** The smallest cross-validation error indicates the most optimal K value. The minimum cross-validation error = 0.56024 which corresponds to K=7.

**Figure E. An *ADMIXTURE* plot showing the proportion of ancestry each hypothetical ancestral population (K = 2) contributes to each studied population.** The mean admixture proportion of each component across each given population was calculated and the sum rescaled to one. * indicates Jewish populations.

**Figure F. An *ADMIXTURE* plot showing the proportion of ancestry each hypothetical ancestral population (K = 3) contributes to each studied population.** The mean admixture proportion of each component across each given population was calculated and the sum rescaled to one. * indicates Jewish populations.

**Figure G. An *ADMIXTURE* plot showing the proportion of ancestry each hypothetical ancestral population (K = 4) contributes to each studied population.** The mean admixture proportion of each component across each given population was calculated and the sum rescaled to one. * indicates Jewish populations.

**Figure H. An *ADMIXTURE* plot showing the proportion of ancestry each hypothetical ancestral population (K = 5) contributes to each studied population.** The mean admixture proportion of each component across each given population was calculated and the sum rescaled to one. * indicates Jewish populations.

**Figure I. An *ADMIXTURE* plot showing the proportion of ancestry each hypothetical ancestral population (K = 6) contributes to each studied population.** The mean admixture proportion of each component across each given population was calculated and the sum rescaled to one. * indicates Jewish populations.

**Figure J. An *ADMIXTURE* plot showing the proportion of ancestry each hypothetical ancestral population (K = 8) contributes to each studied population.** The mean admixture proportion of each component across each given population was calculated and the sum rescaled to one. * indicates Jewish populations.

**Figure K. An *ADMIXTURE* plot showing the proportion of ancestry each hypothetical ancestral population (K = 9) contributes to each studied population.** The mean admixture proportion of each component across each given population was calculated and the sum rescaled to one. * indicates Jewish populations.

**Figure L. An *ADMIXTURE* plot showing the proportion of ancestry each hypothetical ancestral population (K = 10) contributes to each studied population.** The mean admixture proportion of each component across each given population was calculated and the sum rescaled to one. * indicates Jewish populations.

**II. Supporting Tables**

| **Population** | **F_st_** |
| --- | --- |
| Kashmiri Pandit | 0.000 |
| Kshatriya | 0.001 |
| Brahmin | 0.002 |
| Vaish | 0.002 |
| Gujarati | 0.004 |
| Pathan | 0.004 |
| Srivastava | 0.004 |
| Burusho | 0.005 |
| Sindhi | 0.005 |
| Tharu | 0.005 |
| Indian (Jewish) | 0.006 |
| Meghawal | 0.006 |
| Lodi | 0.008 |
| Balochi | 0.009 |
| Brahui | 0.009 |
| Kuruchiyan | 0.009 |
| Gounder | 0.009 |
| Bhil | 0.010 |
| Velama | 0.010 |
| Indian-random (Jewish) | 0.011 |
| Jains | 0.011 |
| Madiga | 0.011 |
| Naidu | 0.011 |
| Mala | 0.012 |
| Minicoy | 0.012 |
| Kurumba | 0.012 |
| Halakki | 0.013 |
| Kamsali | 0.013 |
| Indian-Cochin (Jewish) | 0.014 |
| Adi Dravider | 0.014 |
| Mali | 0.014 |
| Satnami | 0.014 |
| Kurdish | 0.015 |
| Kallar | 0.015 |
| Uygur | 0.016 |
| Adygei | 0.017 |
| Sherpa | 0.017 |
| Hazara | 0.019 |
| Vysya | 0.019 |
| Georgian (Jewish) | 0.020 |
| Romania | 0.020 |
| Gond | 0.020 |
| Sephardi Turkey (Jewish) | 0.021 |
| Hungary | 0.021 |
| Northern Greece | 0.021 |
| Russian | 0.021 |
| Irula | 0.021 |
| Vedda | 0.021 |
| Italy | 0.022 |
| Sephardi Greek (Jewish) | 0.022 |
| Djerbian (Jewish) | 0.022 |
| Serbia | 0.022 |
| Sahariya | 0.022 |
| Ashkenazi (Jewish) | 0.023 |
| Slovenian | 0.023 |
| CEU | 0.023 |
| Syrian (Jewish) | 0.023 |
| Netherlands | 0.023 |
| Portugal | 0.023 |
| Switzerland | 0.023 |
| UK | 0.023 |
| Germany | 0.023 |
| Austria | 0.023 |
| France | 0.023 |
| Czech Republic | 0.023 |
| Iraqi (Jewish) | 0.024 |
| Algeria (Jewish) | 0.024 |
| Denmark | 0.024 |
| Ireland | 0.024 |
| Spain | 0.024 |
| Poland | 0.024 |
| Sweden | 0.024 |
| Palestinian | 0.024 |
| Korku | 0.024 |
| Santhal | 0.024 |
| Morocco (Jewish) | 0.025 |
| Tunisia (Jewish) | 0.025 |
| Norway | 0.025 |
| Munda | 0.025 |
| Iranian (Jewish) | 0.026 |
| Finland | 0.026 |
| Italian (Jewish) | 0.027 |
| Druze | 0.028 |
| Kyrgyzstani | 0.029 |
| Libya (Jewish) | 0.029 |
| Kalash | 0.029 |
| Orcadian | 0.029 |
| Yemen (Jewish) | 0.030 |
| Ho | 0.030 |
| Bhumij | 0.031 |
| Kharia | 0.031 |
| Kattunayakan | 0.032 |
| Basque | 0.033 |
| Malai Kuravar | 0.033 |
| Palliyar | 0.034 |
| Bedouin | 0.037 |
| Sardinian | 0.038 |
| Kashmiri Tibetan | 0.039 |
| Narikuravar | 0.041 |
| Chenchu | 0.041 |
| Birhor | 0.046 |
| Mongolian (Qinghai) | 0.053 |
| Buryat | 0.055 |
| Changpa | 0.059 |
| Tu | 0.060 |
| Cambodian | 0.061 |
| Paniyas | 0.061 |
| Mongolia | 0.063 |
| Tibetan - McLeod Ganj | 0.067 |
| Xibo | 0.067 |
| Tibetan - Qinghai | 0.068 |
| Tibet-refugees | 0.068 |
| Hezhen | 0.070 |
| Oroqen | 0.070 |
| Subba | 0.070 |
| Daur | 0.071 |
| Naxi | 0.073 |
| Yizu | 0.073 |
| Han Chinese | 0.074 |
| Japanese | 0.075 |
| Tujia | 0.075 |
| Dai | 0.077 |
| Miaozu | 0.078 |
| Lahu | 0.079 |
| Nyshi | 0.083 |
| She | 0.084 |
| Aonaga | 0.086 |

**Table A. F_st_ genetic distance values relative to the Kashmiri population in ascending order.**
